# Prenatal diagnosis and early childhood outcome of fetuses with extremely large nuchal translucency

**DOI:** 10.1186/s13039-023-00650-4

**Published:** 2023-09-02

**Authors:** Hang Zhou, Xin Yang, CuiXing Yi, Huizhu Zhong, Simin Yuan, Min Pan, Dongzhi Li, Can Liao

**Affiliations:** 1 Prenatal Diagnostic Center, Guangzhou Women and Children’s Medical Center, Guangzhou Medical University, Guangzhou, Guangdong China; 2 Department of Medical Genetics and Prenatal Diagnosis, Longgang District Maternity and Child Healthcare Hospital of Shenzhen City (Longgang Maternity and Child Institute of Shantou University Medical College), Shenzhen, Guangdong China

**Keywords:** Extremely large nuchal translucency, Cystic hygroma, Hydrops, Prenatal diagnosis, Microarray analysis, Exome sequencing

## Abstract

**Objective:**

To evaluate the prenatal and perinatal outcome of fetuses with extremely large nuchal translucency (eNT) thickness (≥ 6.5 mm).

**Methods:**

193 (0.61%) singleton fetuses with eNT were retrospectively included. Anomaly scan, echocardiography, and chromosomal and genetic test were included in our antenatal investigation. Postnatal follow-up was offered to all newborns.

**Results:**

Major congenital anomalies included congenital heart defect (32.6%, 63/193), hydrops fetalis (13.5%, 26/193), omphalocele (9.3%, 18/193), and skeletal dysplasia (7.8%, 15/193) et al. Abnormal karyotype was identified in 81/115 (70.4%) cases including Turner syndrome (n = 47), Trisomy 18 (n = 17), Trisomy 21 (n = 9), and Trisomy 13 (n = 3). Chromosomal microarray analysis provided informative results with 3.6% (1/28) incremental diagnostic yield over conventional karyotyping. The diagnostic yield of exome sequencing is 10.0% (2/20). There was no significant increase [Odds Ratio (OR) = 1.974; 95% confidence interval 0.863–4.516; *P* = 0.104] in the incidence of chromosomal defects despite the presence of other structural anomalies. Only 13 fetuses were successfully followed up and survived at term, no one was found with developmental delay or mental retardation.

**Conclusions:**

Extremely large NT has a high risk of chromosomal abnormality. CMA and ES improve chromosomal genomic and genetic diagnosis of fetal increased NT. When cytogenetic analysis and morphology assessment are both normal, the outcome is good.

## Introduction

Fetal increased nuchal translucency thickness (NT) has a close association with a chromosomal abnormality, congenital heart disease, various malformations and genetic syndromes [1–3]. The risk of fetal abnormalities increases exponentially with increasing NT thickness measurement. However, there is no significant increase in the risk of an unfavorable outcome above the background population risk when genetic and repeated morphological examinations were normal [4], but some others believed that the fetuses were still at very high risk of poor outcome even when genetic analysis were normal [5]. Scott et al. [6] assessed the outcome of pregnancies with an NT measurement over 6.5 mm in 120 pregnancies, abnormal karyotypes were found in 89 (74.2%) of the group. In 24 pregnancies with known outcomes, six (25%) miscarried, and ten (41.7%) elected termination. In eight surviving children, one case had Noonan’s syndrome, another had cerebral palsy, and the other six children appeared to be entirely normal with follow-up ranging from two to six years. They concluded that, when the karyotype and morphology scan are normal, the outcome is often good despite an extremely large NT.

Some researchers revealed that the presence of septations in fetuses would worsen the prognosis [7], but some others believed that the septations could be visible in all fetuses with increased NT thickness by using an appropriate gain in the transverse plane [8], and they believed that cystic hygroma does not constitute a distinct entity in the first trimester that confers a special risk status independent of the NT thickness.

In this retrospective study, we aimed to evaluate the prenatal outcome of fetuses with extremely large nuchal translucency thickness (≥ 6.5 mm) and perinatal or pediatric outcomes among survivors.

## Materials and methods

This was a retrospective study, a total of 57,732 NT measurements have been performed in Guangzhou Women and Children’s Medical Center between November 2017 and May 2021. During the process of routine NT scan, each woman was provided with written informed consent for future research and only consented images were stored in our database. The study protocol was approved by the Ethics Committee of Guangzhou Women and Children medical center.

Those cases with extremely large fetal NT (≥ 6.5 mm) were included in this study. Images of fetal CRL and NT measurement were retrieved from our database and reviewed by two experienced sonographers followed by the NT measurement quality control standard of the British Fetal Medicine Foundation (FMF). During data review, other morphology anomalies were also collected including septations in a transverse suboccipito-bregmatic section of the fetal head, fetal hydrops, and other structural defects. Cases were assigned into two groups according to whether or not other major defects were observed. The prenatal outcomes of karyotyping, chromosomal microarray analysis (CMA), whole exome sequencing (WES), and birth outcomes were investigated in these two groups.

Invasive prenatal diagnosis was performed by either chorionic villi sampling (CVS) or amniocentesis (AC) after obtaining the informed consent of patients. Karyotyping and/or chromosomal microarray analysis (CMA) was recommended. Anomaly scans at 16–18 and 20–22 weeks together with fetal echocardiography were carried out when they showed normal chromosomes. To investigate the incidence of a genetic syndrome, we reviewed our database and retrieved qualified stored DNA of cases showing normal karyotyping and/or CMA for medical trio exome sequencing (ES) with parental samples [9]. Data for all chromosomal abnormalities, genetic testing, and all live and stillborn infants during the study period was obtained from the database in the Department of Prenatal Diagnosis Center, Guangzhou Women and Children Hospital.

Early childhood outcome was evaluated by hospital record and telephone interviews. Babies delivered in our hospital were followed up through the hospital admission records. In children not followed in our center, a questionnaire was performed by phone including simple questions focused on the infant’s development and divided into five domains: communication, gross motor, fine motor, problem-solving, and personal-social skills.

Statistical analysis was performed using the IBM Statistical program SPSS 25.0. Quantitative data with normal distribution were expressed as means and standards and comparison of continuous data was calculated by unpaired student T-test, otherwise, medians and quartile were expressed and comparison was calculated by Mann–Whitney U test, but the Chi-square test or Fisher exact test was used for categorical data. A P-value of less than 0.05 was considered statistically significant.

## Results

NT scan was performed on 57,732 pregnancies between November 2017 and May 2021, which was found to be 6.5 mm or more in 193 cases (0.33%, 193/57732). The median of maternal age, gestational age, and NT thickness were 30.0 (27.1–33.8) years old, 12.4(range, 11.0 to 13.9) weeks, and 8.7 (6.6–16.4) mm.

The fetal images were successfully assessed and septations were observed in all cases in the transverse suboccipitobregmatic plane of the fetal head. Other major structural defects included congenital heart defect (33.2%, 64/193), hydrops fetalis (13.5%, 26/193), omphalocele (9.3%, 18/193), limb dysplasia (7.8%, 15/193) and holoprosencephaly (4.7%, 9/193) et al.

**Fig. 1:**
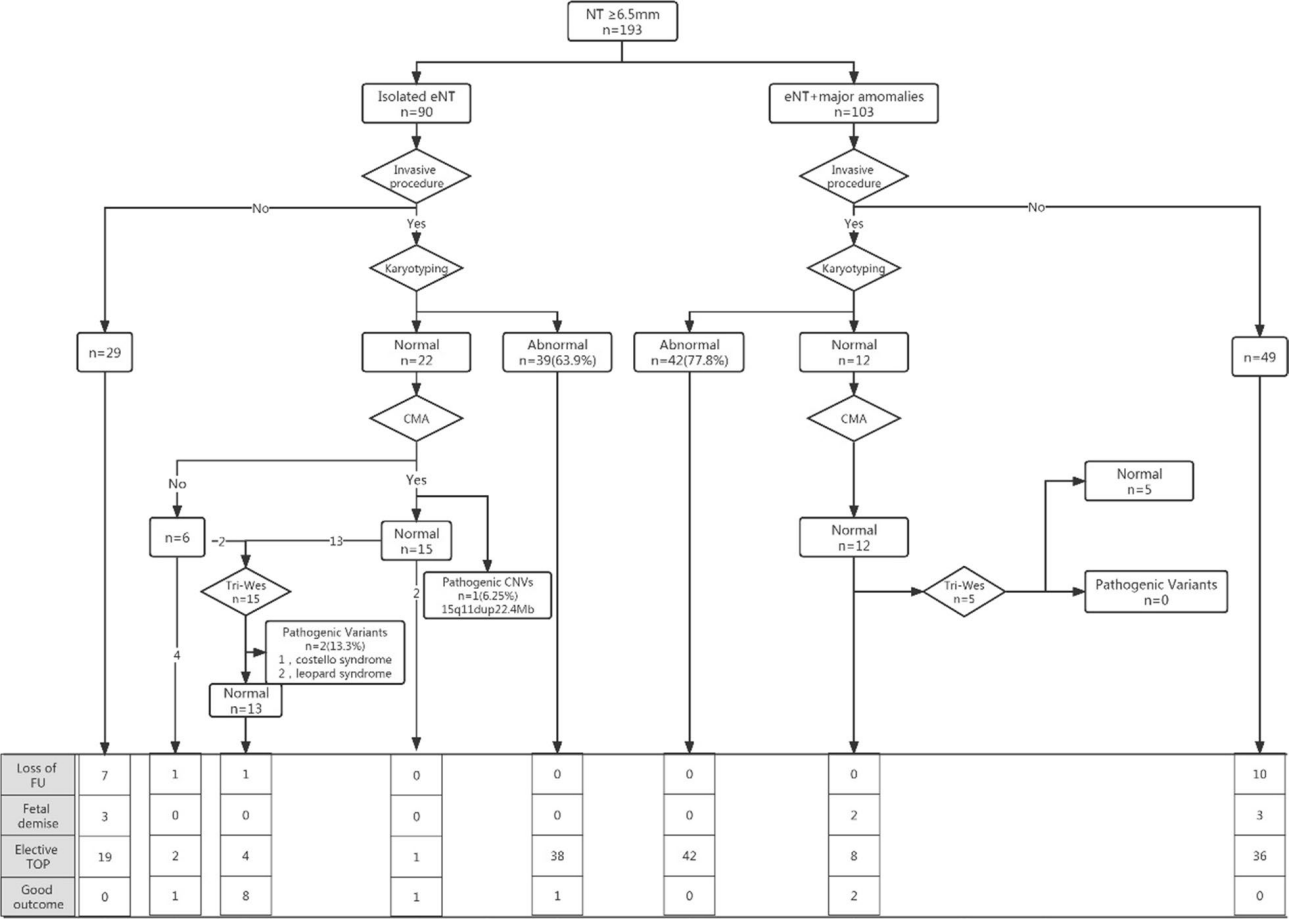
Prenatal outcome of fetuses with extremely large nuchal translucency(eNT) thickness (≥ 6.5 mm) and perinatal or pediatric outcomes among survivors

Among 194 pregnancies with NT ≥ 6.5 mm, the invasive procedure was carried out in 115 (59.3%) cases. Fetal chromosomal results were abnormal in 81(70.4%) cases including 58.0% of Turner syndrome, 21.0% of Trisomy 18, 11.1% of Trisomy 21, and 3.7% of Trisomy 13. In the remaining 34 cases with normal karyotyping, twenty-eight of them were further identified by CMA, which showed pathogenic CNV in 1(3.6%) case identified with microduplication of 15q11.2q21.1 region (15q11.2q21.1 (22,770,421–45,152,371)X3). Tri-exome sequencing (ES) was carried out in 20 cases, two cases (10.0%) were detected to have pathogenic variants including one Costello syndrome and one Leopard syndrome (see Fig. 1). The first case was one with a NT thickness of 11.7 mm, detected with a heterogenous missense mutation of NM_005343.4: c.38G > A (p.Gly13Asp) in HRAS gene, which was reported to lead to Costello syndrome. Another case was a known Leopard syndrome 1 causing variant, NM_002834: c.1530G > C p.(Gln510His), which was found in the PTPN11 gene. The two fetuses with pathogenic variants were diagnosed with isolated eNT without additional structural defects.

In this study, the incidence of abnormal karyotype was 63.9% (39/61) in the isolated eNT group and 77.8% (42/54) in eNT with major congenital anomaly, there is no significant difference between the above two groups (Odds Ratio (OR) = 1.974; 95% confidence interval, 0.863–4.516; *P* = 0.104). For the fetuses with hydrops fetalis, 87.5% (14/16) of cases were found to be with 45, X. For the fetuses with omphalocele, 85.7% (6/7) was Trisomy 18.

In 84 cases with abnormal genetic testing, only one fetus survived who was diagnosed as 47, XXX. For the remaining 31 cases with normal cytogenetic analysis, there were 29 pregnancies with known outcomes. Two cases were found with intrauterine demises and fifteen cases with elective termination. Among twelve survivors (40.0%), ten were born with no structural defects. One fetus with ventricular septal defect (VSD) was delivered at full term and was now six months old and still under regular follow-up. Another hydropic fetus was delivered uneventfully at 38 weeks with pleural effusion resolved at 20 weeks of gestation (see Fig. 1). For 78 cases with no invasive procedure, there were 58 pregnancies with known outcomes. Three intrauterine demises and 55 cases (53.3%) of elective termination (see Fig. 1).

The total percentage of TOPs is 79.8% in this data. The main reasons for the pregnancies to choose TOP are abnormal karyotype (43.5%, 84/193), abnormal ultrasound scan (22.8%, 44/193), and elective termination because of parental request (13.5%, 26/193).

## Discussion

Some studies revealed that the presence of septations in fetuses tends to be worse compared with an isolated increased size of NT [6, 7]. But others believed that septations could be visible in all fetuses with increased NT thickness by using an appropriate gain in the transverse plane by sonographers [8], and they believed that cystic hygroma does not constitute a distinct entity in the first trimester that confers a special risk status independent of the NT thickness. In our study, septations were observed in all cases. It is difficult for us to identify septation as a special risk independent of NT thickness, it seemed that the presence of septation should not play a key role in prenatal counseling. In addition to septation, more than half of cases are associated with other structural malformations including heart malformations, skeletal malformations, omphalocele, and so on, which is consistent with the literature report[10].

In this study, the chromosomal aberration was 70.4%, which is similar to the study of Kagan et al. and Nakamura et al. [11]. There was no statistically significant difference in the incidence of chromosomal aberration no matter if the eNT fetuses were accompanied by other major congenital anomalies, septations or even hydrops. Turner syndrome (45, X) (57.1%) was the most common chromosomal defect, and this proportion increased to 87.5% (14/16) when the fetus was diagnosed with hydrops.

Recently, several groups have reported the potential added value of CMA as an adjunct for conventional karyotyping [12–14]. One meta-analysis [12] which included 17 studies describing CNVs in fetuses with increased NT, showed a 5% (95% CI 0.02–0.08) incremental yield by CMA in isolated and associated increased NT (> 3.5 mm). Maya [13] has reported CMA results in 1588 pregnancies among which fetuses had either normal NT with no other finding or isolated increased NT. For those with NT ≥ 3.5 mm, pathogenic copy number variants were found in 13.8% of cases. However, some other studies [14, 15] believed that there was no obvious contribution of NT thickness to incremental yield by CMA when NT was > 3.5 mm. In this study, CMA yielded an additional diagnostic rate of 3.8% (1/27), which is consistent with the latter conclusion. All in all, our findings supported the recommendation that CMA should be offered for fetuses with NT > 3.5 mm. Of note, for NT ≥ 6.5 mm, the prevalence of the common chromosomal disease(45, X, T21, T18&T13) is much higher (70%) than that of pathogenic CNVs (3.5%).

A recent meta-analysis [16] including 11 studies with data on exome sequencing/genome sequencing(ES/GS) yield including 309 individuals with isolated increased NT > 99th percentile showed 4.9%(15/309) of pathogenic or likely pathogenic variants. Six (40%) of them showed an increased NT of 5 mm or more. In our previous study [17], a genetic syndrome caused by a known disease-causing variant was detected in 1 out of 73(1.4%) fetuses with isolated increased NT(≥ 3.5 mm). In this study, we further performed Tri ES in 20 cases with normal karyotyping and CMA, which included 15 isolated eNT and 5 with other major ultrasound anomalies. Two (10.0%) genetic syndromes were identified including one Costello syndrome and one Leopard syndrome (see Fig. 1). As is known, Noonan syndrome and phenotypically overlapping syndromes such as Costello syndrome and Leopard syndrome are part of the so-called rasopathies and are caused by at least 16 genes, PTPN11 and HRAS are the most prevalent genes. Prenatal features of rasopathies include increased nuchal translucency and/or cystic hygroma, distended jugular lymph sacs, hydrops fetalis, polyhydramnios, pleural effusion, ascites, cardiac defects and renal anomalies. It has been previously estimated that mutations in the rasopathy genes are found in 6.7–19% of fetuses with increased NT and additional anomalies on ultrasound. In our study, rasopathy genes were found in 10% of fetuses. It seemed that the detection of rasopathies genes is very necessary especially when NT is 6.5 mm or more, but it still needs further investigation.

To our knowledge, this was so far the largest retrospective study which included 194 pregnancies with an NT measurement of ≥ 6.5 mm, but only 13 fetuses were followed up successfully and survived at term. Although only a small proportion of the fetuses survived at term, no one with a very poor outcome was identified in this study, which may be due to the small numbers or short time of follow-up. Our result is consistent with the study by Scott et al. [17], which concluded that when the karyotype and morphology scan are both normal in cases, the pregnancy outcome is often good despite an NT measurement above 6.5 mm. Factors that may influence the parents’ decision to terminate the pregnancy were the visual impact of the skin edema around the fetal body, and fearing the possibility of the structural or genetic abnormality undetectable after birth.

Limitations of this study included: (1) Fetuses who were defined with isolated eNT at the first trimester maybe not be really “isolated”, and some ultrasound defects may not be detected in the early gestation. (2) Some of the ultrasound defects could not be confirmed due to the elective TOP. (3) The high rate of terminations may cause the bias of perinatal outcome and healthy survival rate.

In conclusion, extremely large NT has a high risk of chromosomal abnormality, which has no significant increase regardless of the presence of septation or ultrasound malformations. CMA and ES improve chromosomal genomic and genetic diagnosis of fetal increased NT. The outcome will be favorable if cytogenetic analysis and morphology assessment are both normal.

## Data Availability

The data that support the findings of this study are not publicly available due to their containing information that could compromise the privacy of research participants. Further enquiries can be directed to the corresponding author.
